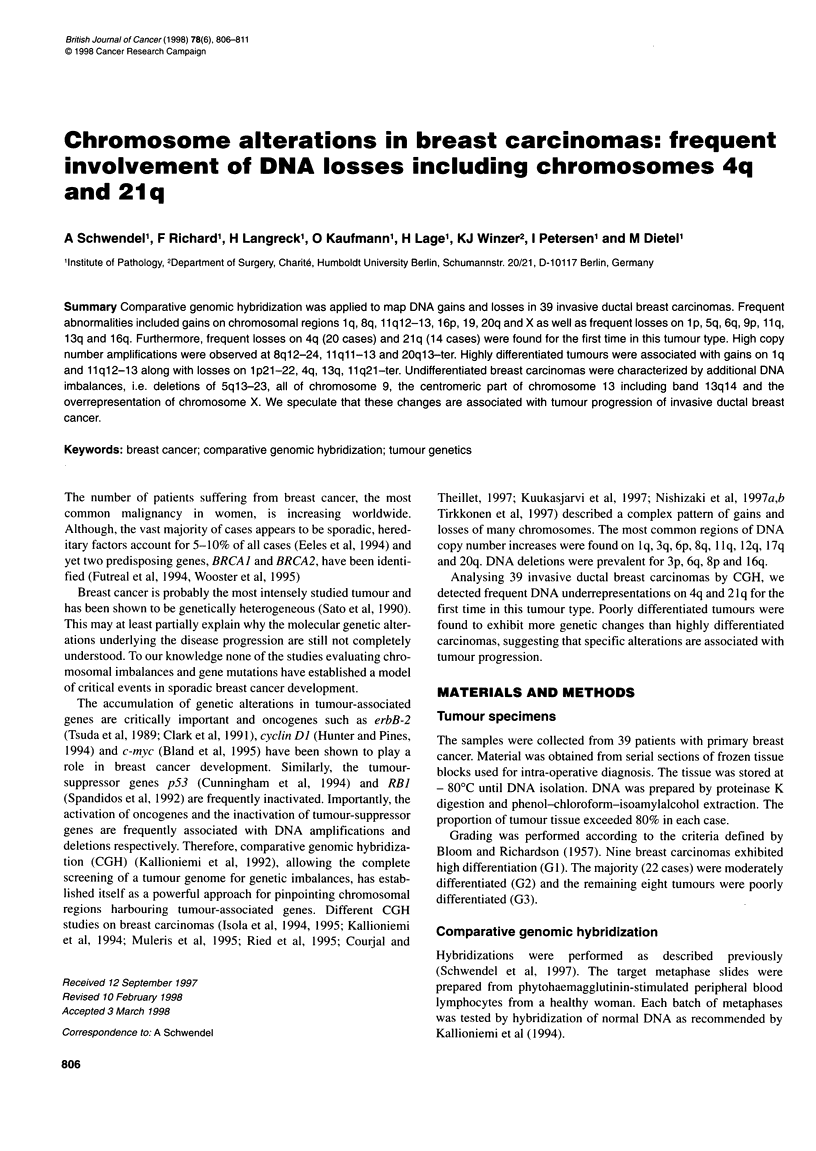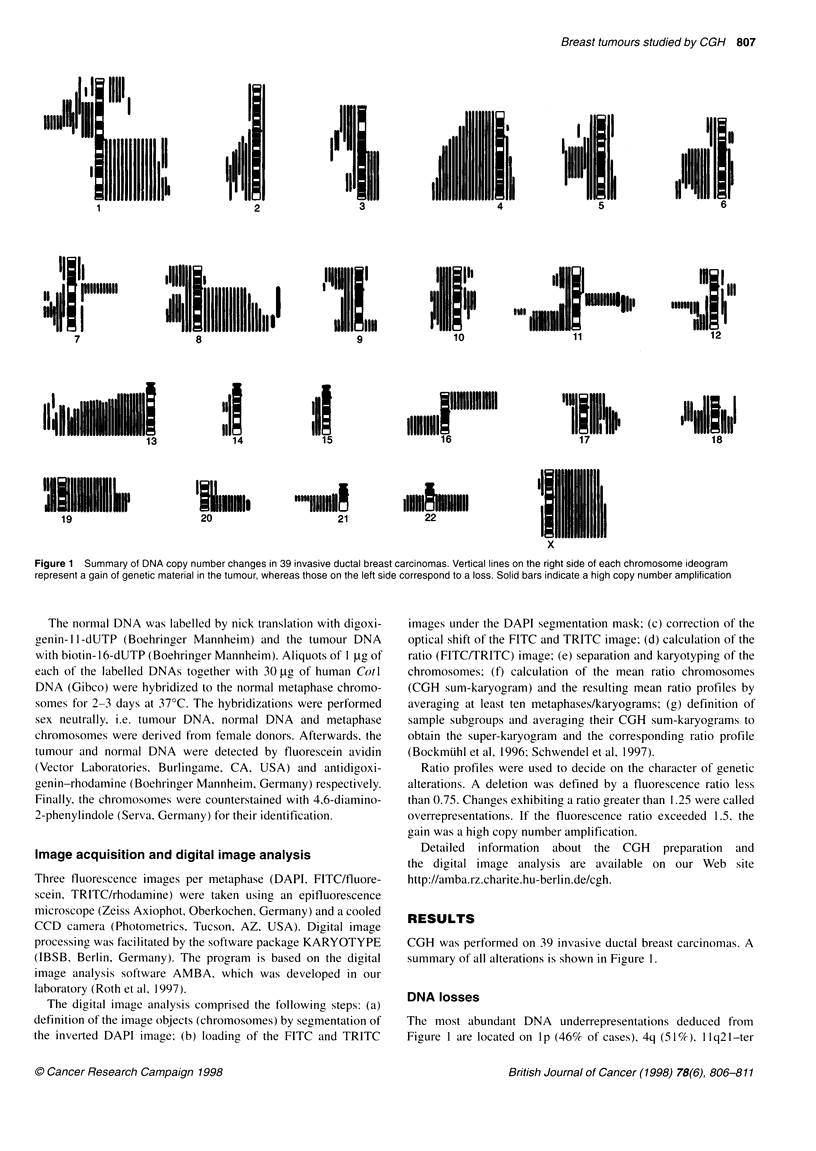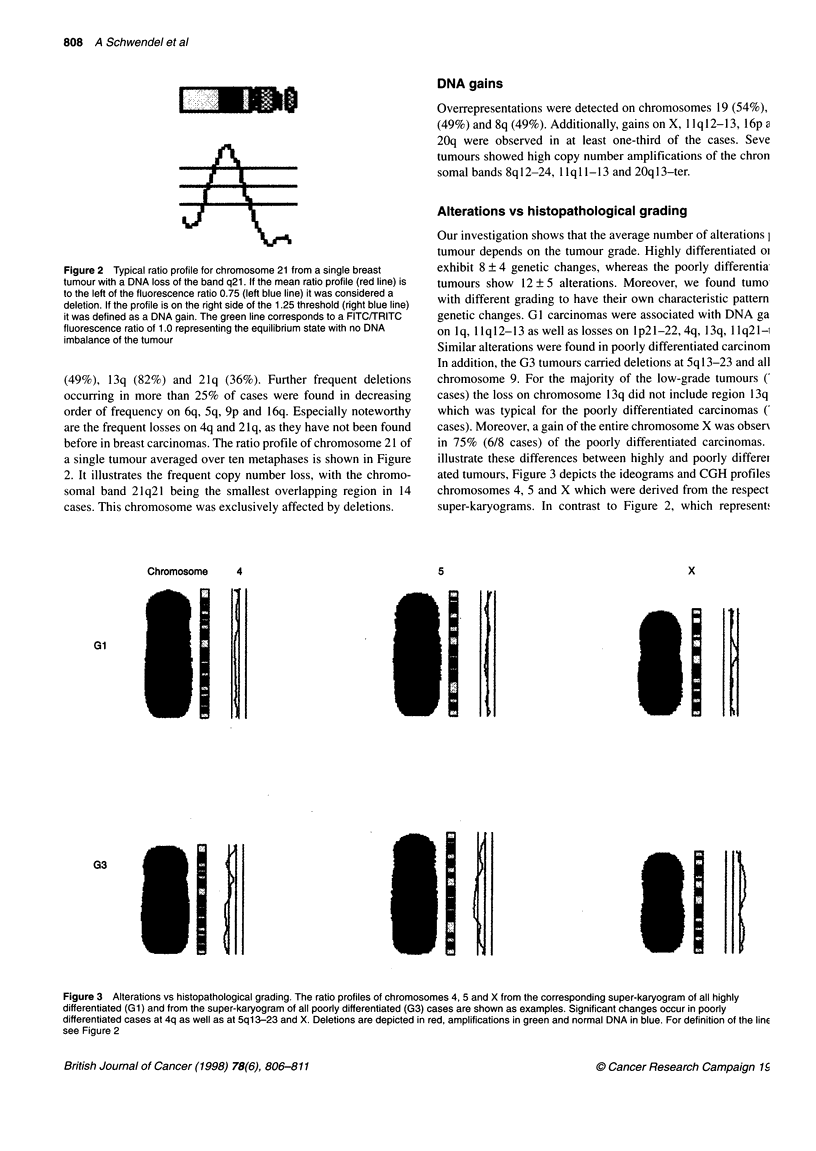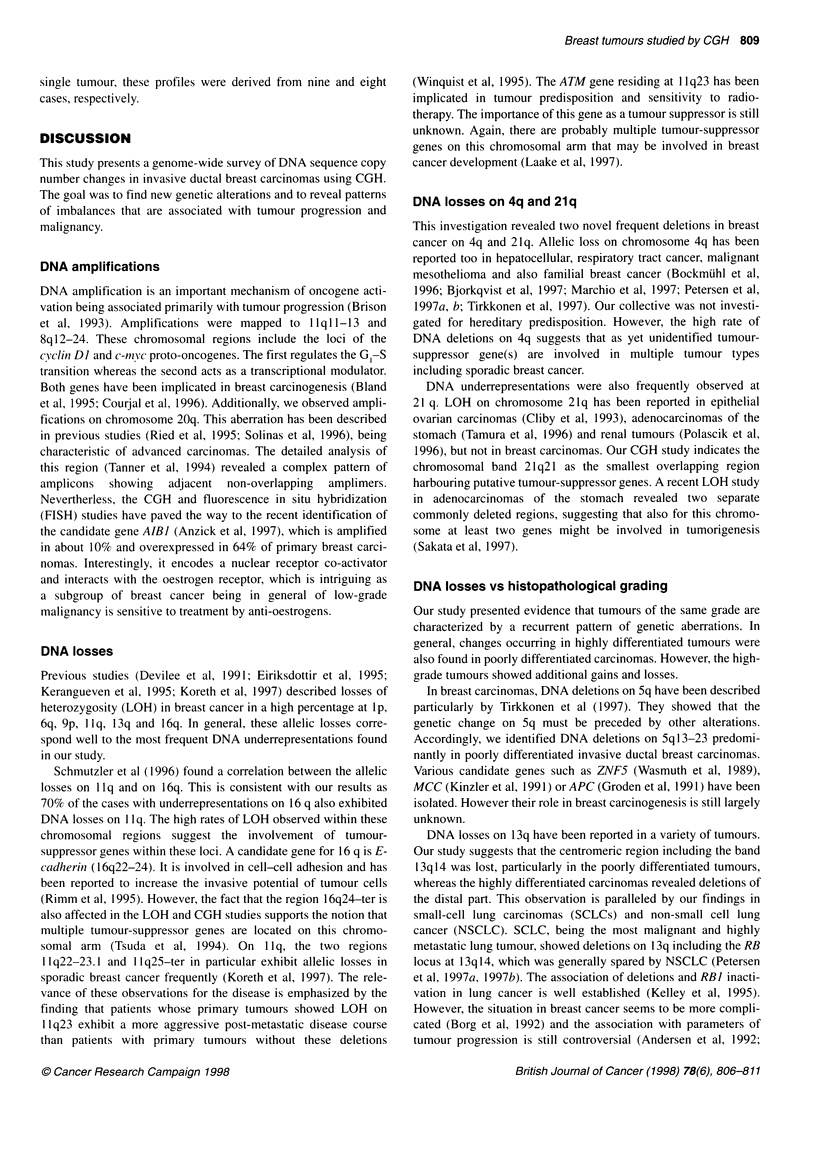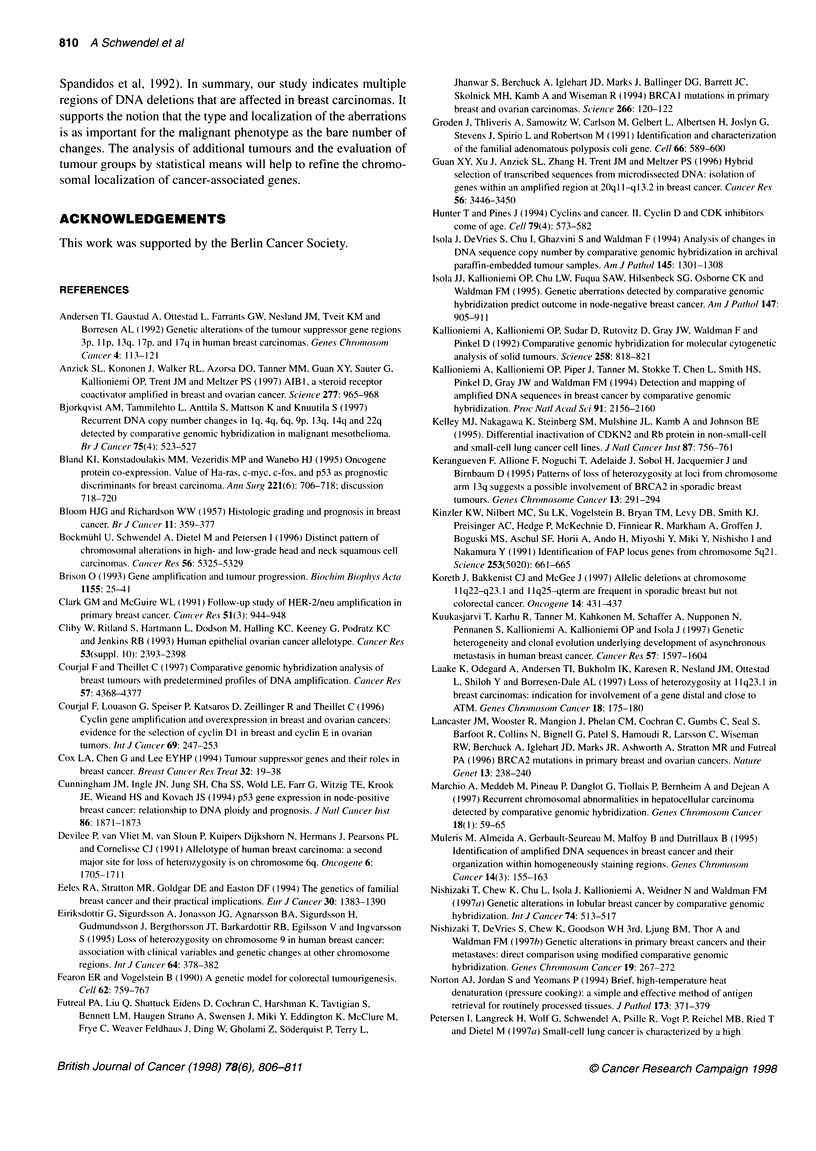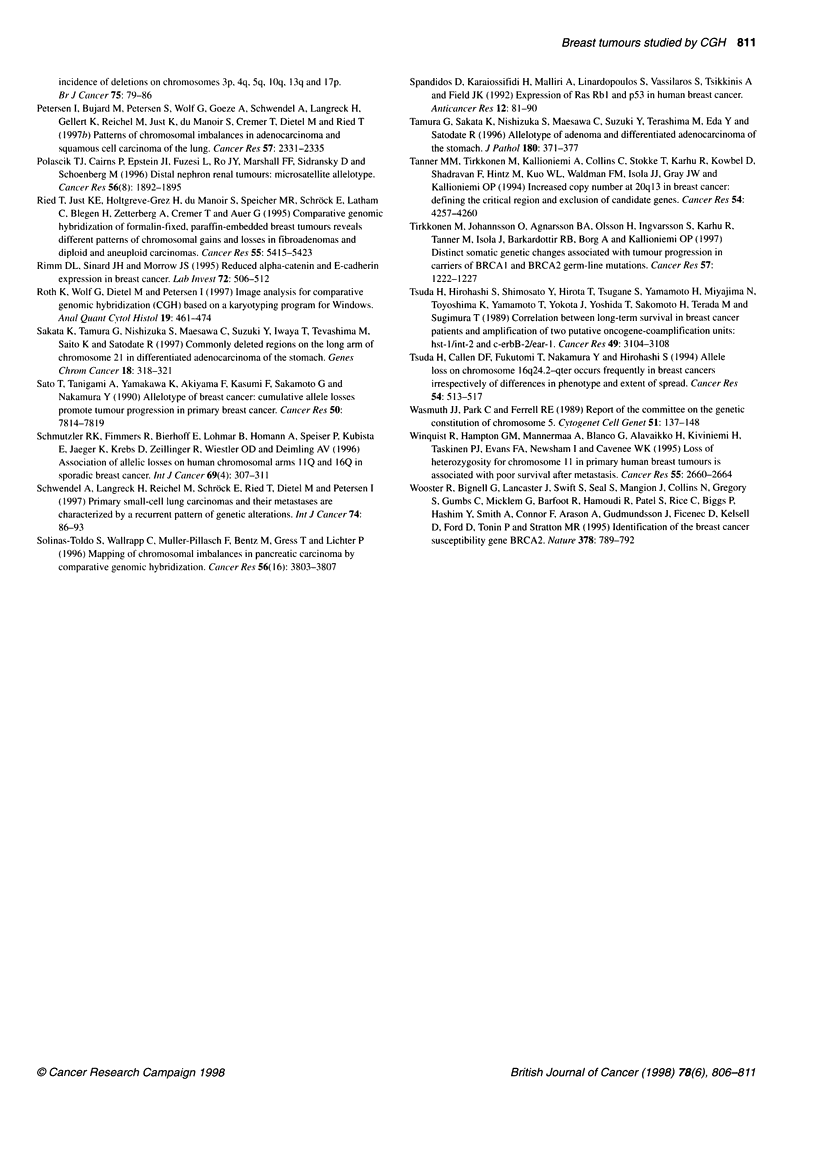# Chromosome alterations in breast carcinomas: frequent involvement of DNA losses including chromosomes 4q and 21q.

**DOI:** 10.1038/bjc.1998.583

**Published:** 1998-09

**Authors:** A. Schwendel, F. Richard, H. Langreck, O. Kaufmann, H. Lage, K. J. Winzer, I. Petersen, M. Dietel

**Affiliations:** Institute of Pathology, Charité, Humboldt University Berlin, Germany.

## Abstract

**Images:**


*British Journal of Cancer* (1998) **78**(6), 806–811
© 1998 Cancer Research Campaign

### Chromosome alterations in breast carcinomas: frequent involvement of DNA losses including chromosomes 4q and 21q

**A Schwendel[1], F Richard[1], H Langreck[1], O Kaufmann[1], H Lage[1], KJ Winzer[2], I Petersen[1] and M Dietel[1]**

[1]Institute of Pathology, [2]Department of Surgery, Charité, Humboldt University Berlin, Schumannstr. 20/21, D-10117 Berlin, Germany

**Summary** Comparative genomic hybridization was applied to map DNA gains and losses in 39 invasive ductal breast carcinomas. Frequent abnormalities included gains on chromosomal regions 1q, 8q, 11q12–13, 16p, 19, 20q and X as well as frequent losses on 1p, 5q, 6q, 9p, 11q, 13q and 16q. Furthermore, frequent losses on 4q (20 cases) and 21q (14 cases) were found for the first time in this tumour type. High copy number amplifications were observed at 8q12–24, 11q11–13 and 20q13–ter. Highly differentiated tumours were associated with gains on 1q and 11q12–13 along with losses on 1p21–22, 4q, 13q, 11q21–ter. Undifferentiated breast carcinomas were characterized by additional DNA imbalances, i.e. deletions of 5q13–23, all of chromosome 9, the centromeric part of chromosome 13 including band 13q14 and the overrepresentation of chromosome X. We speculate that these changes are associated with tumour progression of invasive ductal breast cancer.

**Keywords:** breast cancer; comparative genomic hybridization; tumour genetics

The number of patients suffering from breast cancer, the most common malignancy in women, is increasing worldwide. Although, the vast majority of cases appears to be sporadic, hereditary factors account for 5–10% of all cases (Eeles et al, 1994) and yet two predisposing genes, *BRCA1* and *BRCA2*, have been identified (Futreal et al, 1994, Wooster et al, 1995)

Breast cancer is probably the most intensely studied tumour and has been shown to be genetically heterogeneous (Sato et al, 1990). This may at least partially explain why the molecular genetic alterations underlying the disease progression are still not completely understood. To our knowledge none of the studies evaluating chromosomal imbalances and gene mutations have established a model of critical events in sporadic breast cancer development.

The accumulation of genetic alterations in tumour-associated genes are critically important and oncogenes such as *erbB-2* (Tsuda et al, 1989; Clark et al, 1991), *cyclin D1* (Hunter and Pines, 1994) and *c-myc* (Bland et al, 1995) have been shown to play a role in breast cancer development. Similarly, the tumour-suppressor genes *p53* (Cunningham et al, 1994) and *RB1* (Spandidos et al, 1992) are frequently inactivated. Importantly, the activation of oncogenes and the inactivation of tumour-suppressor genes are frequently associated with DNA amplifications and deletions respectively. Therefore, comparative genomic hybridization (CGH) (Kallioniemi et al, 1992), allowing the complete screening of a tumour genome for genetic imbalances, has established itself as a powerful approach for pinpointing chromosomal regions harbouring tumour-associated genes. Different CGH studies on breast carcinomas (Isola et al, 1994, 1995; Kallioniemi et al, 1994; Muleris et al, 1995; Ried et al, 1995; Courjal and Theillet, 1997; Kuukasjarvi et al, 1997; Nishizaki et al, 1997*a*,*b* Tirkkonen et al, 1997) described a complex pattern of gains and losses of many chromosomes. The most common regions of DNA copy number increases were found on 1q, 3q, 6p, 8q, 11q, 12q, 17q and 20q. DNA deletions were prevalent for 3p, 6q, 8p and 16q.

Analysing 39 invasive ductal breast carcinomas by CGH, we detected frequent DNA underrepresentations on 4q and 21q for the first time in this tumour type. Poorly differentiated tumours were found to exhibit more genetic changes than highly differentiated carcinomas, suggesting that specific alterations are associated with tumour progression.

### MATERIALS AND METHODS

### Tumour specimens

The samples were collected from 39 patients with primary breast cancer. Material was obtained from serial sections of frozen tissue blocks used for intra-operative diagnosis. The tissue was stored at – 80°C until DNA isolation. DNA was prepared by proteinase K digestion and phenol–chloroform–isoamylalcohol extraction. The proportion of tumour tissue exceeded 80% in each case.

Grading was performed according to the criteria defined by Bloom and Richardson (1957). Nine breast carcinomas exhibited high differentiation (G1). The majority (22 cases) were moderately differentiated (G2) and the remaining eight tumours were poorly differentiated (G3).

### Comparative genomic hybridization

Hybridizations were performed as described previously (Schwendel et al, 1997). The target metaphase slides were prepared from phytohaemagglutinin-stimulated peripheral blood lymphocytes from a healthy woman. Each batch of metaphases was tested by hybridization of normal DNA as recommended by Kallioniemi et al (1994).

*Received 12 September 1997*
*Revised 10 February 1998*
*Accepted 3 March 1998*

*Correspondence to:* A Schwendel

**Figure 1** Summary of DNA copy number changes in 39 invasive ductal breast carcinomas. Vertical lines on the right side of each chromosome ideogram represent a gain of genetic material in the tumour, whereas those on the left side correspond to a loss. Solid bars indicate a high copy number amplification

The normal DNA was labelled by nick translation with digoxigenin-11-dUTP (Boehringer Mannheim) and the tumour DNA with biotin-16-dUTP (Boehringer Mannheim). Aliquots of 1 µg of each of the labelled DNAs together with 30 µg of human *Cot*1 DNA (Gibco) were hybridized to the normal metaphase chromosomes for 2–3 days at 37°C. The hybridizations were performed sex neutrally, i.e. tumour DNA, normal DNA and metaphase chromosomes were derived from female donors. Afterwards, the tumour and normal DNA were detected by fluorescein avidin (Vector Laboratories, Burlingame, CA, USA) and antidigoxigenin–rhodamine (Boehringer Mannheim, Germany) respectively. Finally, the chromosomes were counterstained with 4,6-diamino-2-phenylindole (Serva, Germany) for their identification.

### Image acquisition and digital image analysis

Three fluorescence images per metaphase (DAPI, FITC/fluorescein, TRITC/rhodamine) were taken using an epifluorescence microscope (Zeiss Axiophot, Oberkochen, Germany) and a cooled CCD camera (Photometrics, Tucson, AZ, USA). Digital image processing was facilitated by the software package KARYOTYPE (IBSB, Berlin, Germany). The program is based on the digital image analysis software AMBA, which was developed in our laboratory (Roth et al, 1997).

The digital image analysis comprised the following steps: (a) definition of the image objects (chromosomes) by segmentation of the inverted DAPI image; (b) loading of the FITC and TRITC images under the DAPI segmentation mask; (c) correction of the optical shift of the FITC and TRITC image; (d) calculation of the ratio (FITC/TRITC) image; (e) separation and karyotyping of the chromosomes; (f) calculation of the mean ratio chromosomes (CGH sum-karyogram) and the resulting mean ratio profiles by averaging at least ten metaphases/karyograms; (g) definition of sample subgroups and averaging their CGH sum-karyograms to obtain the super-karyogram and the corresponding ratio profile (Bockmühl et al, 1996; Schwendel et al, 1997).

Ratio profiles were used to decide on the character of genetic alterations. A deletion was defined by a fluorescence ratio less than 0.75. Changes exhibiting a ratio greater than 1.25 were called overrepresentations. If the fluorescence ratio exceeded 1.5, the gain was a high copy number amplification.

Detailed information about the CGH preparation and the digital image analysis are available on our Web site http://amba.rz.charite.hu-berlin.de/cgh.

### RESULTS

CGH was performed on 39 invasive ductal breast carcinomas. A summary of all alterations is shown in Figure 1.

### DNA losses

The most abundant DNA underrepresentations deduced from Figure 1 are located on 1p (46% of cases), 4q (51%), 11q21–ter

© Cancer Research Campaign 1998

**Figure 2** Typical ratio profile for chromosome 21 from a single breast tumour with a DNA loss of the band q21. If the mean ratio profile (red line) is to the left of the fluorescence ratio 0.75 (left blue line) it was considered a deletion. If the profile is on the right side of the 1.25 threshold (right blue line) it was defined as a DNA gain. The green line corresponds to a FITC/TRITC fluorescence ratio of 1.0 representing the equilibrium state with no DNA imbalance of the tumour

(49%), 13q (82%) and 21q (36%). Further frequent deletions occurring in more than 25% of cases were found in decreasing order of frequency on 6q, 5q, 9p and 16q. Especially noteworthy are the frequent losses on 4q and 21q, as they have not been found before in breast carcinomas. The ratio profile of chromosome 21 of a single tumour averaged over ten metaphases is shown in Figure 2. It illustrates the frequent copy number loss, with the chromosomal band 21q21 being the smallest overlapping region in 14 cases. This chromosome was exclusively affected by deletions.

### DNA gains

Overrepresentations were detected on chromosomes 19 (54%), (49%) and 8q (49%). Additionally, gains on X, 11q12–13, 16p a 20q were observed in at least one-third of the cases. Seve tumours showed high copy number amplifications of the chron somal bands 8q12–24, 11q11–13 and 20q13–ter.

### Alterations vs histopathological grading

Our investigation shows that the average number of alterations | tumour depends on the tumour grade. Highly differentiated oı exhibit 8 ± 4 genetic changes, whereas the poorly differentia tumours show 12 ± 5 alterations. Moreover, we found tumo with different grading to have their own characteristic pattern genetic changes. G1 carcinomas were associated with DNA ga on 1q, 11q12–13 as well as losses on 1p21–22, 4q, 13q, 11q21–ı Similar alterations were found in poorly differentiated carcinom In addition, the G3 tumours carried deletions at 5q13–23 and all chromosome 9. For the majority of the low-grade tumours (' cases) the loss on chromosome 13q did not include region 13q which was typical for the poorly differentiated carcinomas (' cases). Moreover, a gain of the entire chromosome X was obser\ in 75% (6/8 cases) of the poorly differentiated carcinomas. illustrate these differences between highly and poorly differeı ated tumours, Figure 3 depicts the ideograms and CGH profiles chromosomes 4, 5 and X which were derived from the respect super-karyograms. In contrast to Figure 2, which represents

**Figure 3** Alterations vs histopathological grading. The ratio profiles of chromosomes 4, 5 and X from the corresponding super-karyogram of all highly differentiated (G1) and from the super-karyogram of all poorly differentiated (G3) cases are shown as examples. Significant changes occur in poorly differentiated cases at 4q as well as at 5q13–23 and X. Deletions are depicted in red, amplifications in green and normal DNA in blue. For definition of the line see Figure 2

*© Cancer Research Campaign 1ς*

single tumour, these profiles were derived from nine and eight cases, respectively.

### DISCUSSION

This study presents a genome-wide survey of DNA sequence copy number changes in invasive ductal breast carcinomas using CGH. The goal was to find new genetic alterations and to reveal patterns of imbalances that are associated with tumour progression and malignancy.

### DNA amplifications

DNA amplification is an important mechanism of oncogene activation being associated primarily with tumour progression (Brison et al, 1993). Amplifications were mapped to 11q11–13 and 8q12–24. These chromosomal regions include the loci of the *cyclin D1* and *c-myc* proto-oncogenes. The first regulates the $G_1$–S transition whereas the second acts as a transcriptional modulator. Both genes have been implicated in breast carcinogenesis (Bland et al, 1995; Courjal et al, 1996). Additionally, we observed amplifications on chromosome 20q. This aberration has been described in previous studies (Ried et al, 1995; Solinas et al, 1996), being characteristic of advanced carcinomas. The detailed analysis of this region (Tanner et al, 1994) revealed a complex pattern of amplicons showing adjacent non-overlapping amplimers. Nevertheless, the CGH and fluorescence in situ hybridization (FISH) studies have paved the way to the recent identification of the candidate gene *AIB1* (Anzick et al, 1997), which is amplified in about 10% and overexpressed in 64% of primary breast carcinomas. Interestingly, it encodes a nuclear receptor co-activator and interacts with the oestrogen receptor, which is intriguing as a subgroup of breast cancer being in general of low-grade malignancy is sensitive to treatment by anti-oestrogens.

### DNA losses

Previous studies (Devilee et al, 1991; Eiriksdottir et al, 1995; Kerangueven et al, 1995; Koreth et al, 1997) described losses of heterozygosity (LOH) in breast cancer in a high percentage at 1p, 6q, 9p, 11q, 13q and 16q. In general, these allelic losses correspond well to the most frequent DNA underrepresentations found in our study.

Schmutzler et al (1996) found a correlation between the allelic losses on 11q and on 16q. This is consistent with our results as 70% of the cases with underrepresentations on 16 q also exhibited DNA losses on 11q. The high rates of LOH observed within these chromosomal regions suggest the involvement of tumour-suppressor genes within these loci. A candidate gene for 16 q is *E-cadherin* (16q22–24). It is involved in cell–cell adhesion and has been reported to increase the invasive potential of tumour cells (Rimm et al, 1995). However, the fact that the region 16q24–ter is also affected in the LOH and CGH studies supports the notion that multiple tumour-suppressor genes are located on this chromosomal arm (Tsuda et al, 1994). On 11q, the two regions 11q22–23.1 and 11q25–ter in particular exhibit allelic losses in sporadic breast cancer frequently (Koreth et al, 1997). The relevance of these observations for the disease is emphasized by the finding that patients whose primary tumours showed LOH on 11q23 exhibit a more aggressive post-metastatic disease course than patients with primary tumours without these deletions (Winquist et al, 1995). The *ATM* gene residing at 11q23 has been implicated in tumour predisposition and sensitivity to radiotherapy. The importance of this gene as a tumour suppressor is still unknown. Again, there are probably multiple tumour-suppressor genes on this chromosomal arm that may be involved in breast cancer development (Laake et al, 1997).

### DNA losses on 4q and 21q

This investigation revealed two novel frequent deletions in breast cancer on 4q and 21q. Allelic loss on chromosome 4q has been reported too in hepatocellular, respiratory tract cancer, malignant mesothelioma and also familial breast cancer (Bockmühl et al, 1996; Bjorkqvist et al, 1997; Marchio et al, 1997; Petersen et al, 1997*a*, *b*; Tirkkonen et al, 1997). Our collective was not investigated for hereditary predisposition. However, the high rate of DNA deletions on 4q suggests that as yet unidentified tumour-suppressor gene(s) are involved in multiple tumour types including sporadic breast cancer.

DNA underrepresentations were also frequently observed at 21 q. LOH on chromosome 21q has been reported in epithelial ovarian carcinomas (Cliby et al, 1993), adenocarcinomas of the stomach (Tamura et al, 1996) and renal tumours (Polascik et al, 1996), but not in breast carcinomas. Our CGH study indicates the chromosomal band 21q21 as the smallest overlapping region harbouring putative tumour-suppressor genes. A recent LOH study in adenocarcinomas of the stomach revealed two separate commonly deleted regions, suggesting that also for this chromosome at least two genes might be involved in tumorigenesis (Sakata et al, 1997).

### DNA losses vs histopathological grading

Our study presented evidence that tumours of the same grade are characterized by a recurrent pattern of genetic aberrations. In general, changes occurring in highly differentiated tumours were also found in poorly differentiated carcinomas. However, the high-grade tumours showed additional gains and losses.

In breast carcinomas, DNA deletions on 5q have been described particularly by Tirkkonen et al (1997). They showed that the genetic change on 5q must be preceded by other alterations. Accordingly, we identified DNA deletions on 5q13–23 predominantly in poorly differentiated invasive ductal breast carcinomas. Various candidate genes such as *ZNF5* (Wasmuth et al, 1989), *MCC* (Kinzler et al, 1991) or *APC* (Groden et al, 1991) have been isolated. However their role in breast carcinogenesis is still largely unknown.

DNA losses on 13q have been reported in a variety of tumours. Our study suggests that the centromeric region including the band 13q14 was lost, particularly in the poorly differentiated tumours, whereas the highly differentiated carcinomas revealed deletions of the distal part. This observation is paralleled by our findings in small-cell lung carcinomas (SCLCs) and non-small cell lung cancer (NSCLC). SCLC, being the most malignant and highly metastatic lung tumour, showed deletions on 13q including the *RB* locus at 13q14, which was generally spared by NSCLC (Petersen et al, 1997*a*, 1997*b*). The association of deletions and *RB1* inactivation in lung cancer is well established (Kelley et al, 1995). However, the situation in breast cancer seems to be more complicated (Borg et al, 1992) and the association with parameters of tumour progression is still controversial (Andersen et al, 1992;

© Cancer Research Campaign 1998

Spandidos et al, 1992). In summary, our study indicates multiple regions of DNA deletions that are affected in breast carcinomas. It supports the notion that the type and localization of the aberrations is as important for the malignant phenotype as the bare number of changes. The analysis of additional tumours and the evaluation of tumour groups by statistical means will help to refine the chromosomal localization of cancer-associated genes.

### ACKNOWLEDGEMENTS

This work was supported by the Berlin Cancer Society.

### REFERENCES

Andersen TI, Gaustad A, Ottestad L, Farrants GW, Nesland JM, Tveit KM and Borresen AL (1992) Genetic alterations of the tumour suppressor gene regions 3p, 11p, 13q, 17p, and 17q in human breast carcinomas. *Genes Chromosom Cancer* **4**: 113–121

Anzick SL, Kononen J, Walker RL, Azorsa DO, Tanner MM, Guan XY, Sauter G, Kallioniemi OP, Trent JM and Meltzer PS (1997) AIB1, a steroid receptor coactivator amplified in breast and ovarian cancer. *Science* **277**: 965–968

Bjorkqvist AM, Tammilehto L, Anttila S, Mattson K and Knuutila S (1997) Recurrent DNA copy number changes in 1q, 4q, 6q, 9p, 13q, 14q and 22q detected by comparative genomic hybridization in malignant mesothelioma. *Br J Cancer* **75**(4): 523–527

Bland KI, Konstadoulakis MM, Vezeridis MP and Wanebo HJ (1995) Oncogene protein co-expression. Value of Ha-ras, c-myc, c-fos, and p53 as prognostic discriminants for breast carcinoma. *Ann Surg* **221**(6): 706–718; discussion 718–720

Bloom HJG and Richardson WW (1957) Histologic grading and prognosis in breast cancer. *Br J Cancer* **11**: 359–377

Bockmühl U, Schwendel A, Dietel M and Petersen I (1996) Distinct pattern of chromosomal alterations in high- and low-grade head and neck squamous cell carcinomas. *Cancer Res* **56**: 5325–5329

Brison O (1993) Gene amplification and tumour progression. *Biochim Biophys Acta* **1155**: 25–41

Clark GM and McGuire WL (1991) Follow-up study of HER-2/neu amplification in primary breast cancer. *Cancer Res* **51**(3): 944–948

Cliby W, Ritland S, Hartmann L, Dodson M, Halling KC, Keeney G, Podratz KC and Jenkins RB (1993) Human epithelial ovarian cancer allelotype. *Cancer Res* **53**(suppl. 10): 2393–2398

Courjal F and Theillet C (1997) Comparative genomic hybridization analysis of breast tumours with predetermined profiles of DNA amplification. *Cancer Res* **57**: 4368–4377

Courjal F, Louason G, Speiser P, Katsaros D, Zeillinger R and Theillet C (1996) Cyclin gene amplification and overexpression in breast and ovarian cancers: evidence for the selection of cyclin D1 in breast and cyclin E in ovarian tumors. *Int J Cancer* **69**: 247–253

Cox LA, Chen G and Lee EYHP (1994) Tumour suppressor genes and their roles in breast cancer. *Breast Cancer Res Treat* **32**: 19–38

Cunningham JM, Ingle JN, Jung SH, Cha SS, Wold LE, Farr G, Witzig TE, Krook JE, Wieand HS and Kovach JS (1994) p53 gene expression in node-positive breast cancer: relationship to DNA ploidy and prognosis. *J Natl Cancer Inst* **86**: 1871–1873

Devilee P, van Vliet M, van Sloun P, Kuipers Dijkshorn N, Hermans J, Pearsons PL and Cornelisse CJ (1991) Allelotype of human breast carcinoma: a second major site for loss of heterozygosity is on chromosome 6q. *Oncogene* **6**: 1705–1711

Eeles RA, Stratton MR, Goldgar DE and Easton DF (1994) The genetics of familial breast cancer and their practical implications. *Eur J Cancer* **30**: 1383–1390

Eiriksdottir G, Sigurdsson A, Jonasson JG, Agnarsson BA, Sigurdsson H, Gudmundsson J, Bergthorsson JT, Barkardottir RB, Egilsson V and Ingvarsson S (1995) Loss of heterozygosity on chromosome 9 in human breast cancer: association with clinical variables and genetic changes at other chromosome regions. *Int J Cancer* **64**: 378–382

Fearon ER and Vogelstein B (1990) A genetic model for colorectal tumourigenesis. *Cell* **62**: 759–767

Futreal PA, Liu Q, Shattuck Eidens D, Cochran C, Harshman K, Tavtigian S, Bennett LM, Haugen Strano A, Swensen J, Miki Y, Eddington K, McClure M, Frye C, Weaver Feldhaus J, Ding W, Gholami Z, Söderquist P, Terry L, Jhanwar S, Berchuck A, Iglehart JD, Marks J, Ballinger DG, Barrett JC, Skolnick MH, Kamb A and Wiseman R (1994) BRCA1 mutations in primary breast and ovarian carcinomas. *Science* **266**: 120–122

Groden J, Thliveris A, Samowitz W, Carlson M, Gelbert L, Albertsen H, Joslyn G, Stevens J, Spirio L and Robertson M (1991) Identification and characterization of the familial adenomatous polyposis coli gene. *Cell* **66**: 589–600

Guan XY, Xu J, Anzick SL, Zhang H, Trent JM and Meltzer PS (1996) Hybrid selection of transcribed sequences from microdissected DNA: isolation of genes within an amplified region at 20q11–q13.2 in breast cancer. *Cancer Res* **56**: 3446–3450

Hunter T and Pines J (1994) Cyclins and cancer. II. Cyclin D and CDK inhibitors come of age. *Cell* **79**(4): 573–582

Isola J, DeVries S, Chu I, Ghazvini S and Waldman F (1994) Analysis of changes in DNA sequence copy number by comparative genomic hybridization in archival paraffin-embedded tumour samples. *Am J Pathol* **145**: 1301–1308

Isola JJ, Kallioniemi OP, Chu LW, Fuqua SAW, Hilsenbeck SG, Osborne CK and Waldman FM (1995). Genetic aberrations detected by comparative genomic hybridization predict outcome in node-negative breast cancer. *Am J Pathol* **147**: 905–911

Kallioniemi A, Kallioniemi OP, Sudar D, Rutovitz D, Gray JW, Waldman F and Pinkel D (1992) Comparative genomic hybridization for molecular cytogenetic analysis of solid tumours. *Science* **258**: 818–821

Kallioniemi A, Kallioniemi OP, Piper J, Tanner M, Stokke T, Chen L, Smith HS, Pinkel D, Gray JW and Waldman FM (1994) Detection and mapping of amplified DNA sequences in breast cancer by comparative genomic hybridization. *Proc Natl Acad Sci* **91**: 2156–2160

Kelley MJ, Nakagawa K, Steinberg SM, Mulshine JL, Kamb A and Johnson BE (1995). Differential inactivation of CDKN2 and Rb protein in non-small-cell and small-cell lung cancer cell lines. *J Natl Cancer Inst* **87**: 756–761

Kerangueven F, Allione F, Noguchi T, Adelaide J, Sobol H, Jacquemier J and Birnbaum D (1995) Patterns of loss of heterozygosity at loci from chromosome arm 13q suggests a possible involvement of BRCA2 in sporadic breast tumours. *Genes Chromosome Cancer* **13**: 291–294

Kinzler KW, Nilbert MC, Su LK, Vogelstein B, Bryan TM, Levy DB, Smith KJ, Preisinger AC, Hedge P, McKechnie D, Finniear R, Markham A, Groffen J, Boguski MS, Aschul SF, Horii A, Ando H, Miyoshi Y, Miki Y, Nishisho I and Nakamura Y (1991) Identification of FAP locus genes from chromosome 5q21. *Science* **253**(5020): 661–665

Koreth J, Bakkenist CJ and McGee J (1997) Allelic deletions at chromosome 11q22–q23.1 and 11q25–qterm are frequent in sporadic breast but not colorectal cancer. *Oncogene* **14**: 431–437

Kuukasjarvi T, Karhu R, Tanner M, Kahkonen M, Schaffer A, Nupponen N, Pennanen S, Kallioniemi A, Kallioniemi OP and Isola J (1997) Genetic heterogeneity and clonal evolution underlying development of asynchronous metastasis in human breast cancer. *Cancer Res* **57**: 1597–1604

Laake K, Odegard A, Andersen TI, Bukholm IK, Karesen R, Nesland JM, Ottestad L, Shiloh Y and Borresen-Dale AL (1997) Loss of heterozygosity at 11q23.1 in breast carcinomas: indication for involvement of a gene distal and close to ATM. *Genes Chromosom Cancer* **18**: 175–180

Lancaster JM, Wooster R, Mangion J, Phelan CM, Cochran C, Gumbs C, Seal S, Barfoot R, Collins N, Bignell G, Patel S, Hamoudi R, Larsson C, Wiseman RW, Berchuck A, Iglehart JD, Marks JR, Ashworth A, Stratton MR and Futreal PA (1996) BRCA2 mutations in primary breast and ovarian cancers. *Nature Genet* **13**: 238–240

Marchio A, Meddeb M, Pineau P, Danglot G, Tiollais P, Bernheim A and Dejean A (1997) Recurrent chromosomal abnormalities in hepatocellular carcinoma detected by comparative genomic hybridization. *Genes Chromosom Cancer* **18**(1): 59–65

Muleris M, Almeida A, Gerbault-Seureau M, Malfoy B and Dutrillaux B (1995) Identification of amplified DNA sequences in breast cancer and their organization within homogeneously staining regions. *Genes Chromosom Cancer* **14**(3): 155–163

Nishizaki T, Chew K, Chu L, Isola J, Kallioniemi A, Weidner N and Waldman FM (1997*a*) Genetic alterations in lobular breast cancer by comparative genomic hybridization. *Int J Cancer* **74**: 513–517

Nishizaki T, DeVries S, Chew K, Goodson WH 3rd, Ljung BM, Thor A and Waldman FM (1997*b*) Genetic alterations in primary breast cancers and their metastases: direct comparison using modified comparative genomic hybridization. *Genes Chromosom Cancer* **19**: 267–272

Norton AJ, Jordan S and Yeomans P (1994) Brief, high-temperature heat denaturation (pressure cooking): a simple and effective method of antigen retrieval for routinely processed tissues. *J Pathol* **173**: 371–379

Petersen I, Langreck H, Wolf G, Schwendel A, Psille R, Vogt P, Reichel MB, Ried T and Dietel M (1997*a*) Small-cell lung cancer is characterized by a high

© Cancer Research Campaign 1998

incidence of deletions on chromosomes 3p, 4q, 5q, 10q, 13q and 17p. *Br J Cancer* **75**: 79–86

Petersen I, Bujard M, Petersen S, Wolf G, Goeze A, Schwendel A, Langreck H, Gellert K, Reichel M, Just K, du Manoir S, Cremer T, Dietel M and Ried T (1997*b*) Patterns of chromosomal imbalances in adenocarcinoma and squamous cell carcinoma of the lung. *Cancer Res* **57**: 2331–2335

Polascik TJ, Cairns P, Epstein JI, Fuzesi L, Ro JY, Marshall FF, Sidransky D and Schoenberg M (1996) Distal nephron renal tumours: microsatellite allelotype. *Cancer Res* **56**(8): 1892–1895

Ried T, Just KE, Holtgreve-Grez H, du Manoir S, Speicher MR, Schröck E, Latham C, Blegen H, Zetterberg A, Cremer T and Auer G (1995) Comparative genomic hybridization of formalin-fixed, paraffin-embedded breast tumours reveals different patterns of chromosomal gains and losses in fibroadenomas and diploid and aneuploid carcinomas. *Cancer Res* **55**: 5415–5423

Rimm DL, Sinard JH and Morrow JS (1995) Reduced alpha-catenin and E-cadherin expression in breast cancer. *Lab Invest* **72**: 506–512

Roth K, Wolf G, Dietel M and Petersen I (1997) Image analysis for comparative genomic hybridization (CGH) based on a karyotyping program for Windows. *Anal Quant Cytol Histol* **19**: 461–474

Sakata K, Tamura G, Nishizuka S, Maesawa C, Suzuki Y, Iwaya T, Tevashima M, Saito K and Satodate R (1997) Commonly deleted regions on the long arm of chromosome 21 in differentiated adenocarcinoma of the stomach. *Genes Chrom Cancer* **18**: 318–321

Sato T, Tanigami A, Yamakawa K, Akiyama F, Kasumi F, Sakamoto G and Nakamura Y (1990) Allelotype of breast cancer: cumulative allele losses promote tumour progression in primary breast cancer. *Cancer Res* **50**: 7814–7819

Schmutzler RK, Fimmers R, Bierhoff E, Lohmar B, Homann A, Speiser P, Kubista E, Jaeger K, Krebs D, Zeillinger R, Wiestler OD and Deimling AV (1996) Association of allelic losses on human chromosomal arms 11Q and 16Q in sporadic breast cancer. *Int J Cancer* **69**(4): 307–311

Schwendel A, Langreck H, Reichel M, Schröck E, Ried T, Dietel M and Petersen I (1997) Primary small-cell lung carcinomas and their metastases are characterized by a recurrent pattern of genetic alterations. *Int J Cancer* **74**: 86–93

Solinas-Toldo S, Wallrapp C, Muller-Pillasch F, Bentz M, Gress T and Lichter P (1996) Mapping of chromosomal imbalances in pancreatic carcinoma by comparative genomic hybridization. *Cancer Res* **56**(16): 3803–3807

Spandidos D, Karaiossifidi H, Malliri A, Linardopoulos S, Vassilaros S, Tsikkinis A and Field JK (1992) Expression of Ras Rb1 and p53 in human breast cancer. *Anticancer Res* **12**: 81–90

Tamura G, Sakata K, Nishizuka S, Maesawa C, Suzuki Y, Terashima M, Eda Y and Satodate R (1996) Allelotype of adenoma and differentiated adenocarcinoma of the stomach. *J Pathol* **180**: 371–377

Tanner MM, Tirkkonen M, Kallioniemi A, Collins C, Stokke T, Karhu R, Kowbel D, Shadravan F, Hintz M, Kuo WL, Waldman FM, Isola JJ, Gray JW and Kallioniemi OP (1994) Increased copy number at 20q13 in breast cancer: defining the critical region and exclusion of candidate genes. *Cancer Res* **54**: 4257–4260

Tirkkonen M, Johannsson O, Agnarsson BA, Olsson H, Ingvarsson S, Karhu R, Tanner M, Isola J, Barkardottir RB, Borg A and Kallioniemi OP (1997) Distinct somatic genetic changes associated with tumour progression in carriers of BRCA1 and BRCA2 germ-line mutations. *Cancer Res* **57**: 1222–1227

Tsuda H, Hirohashi S, Shimosato Y, Hirota T, Tsugane S, Yamamoto H, Miyajima N, Toyoshima K, Yamamoto T, Yokota J, Yoshida T, Sakomoto H, Terada M and Sugimura T (1989) Correlation between long-term survival in breast cancer patients and amplification of two putative oncogene-coamplification units: hst-1/int-2 and c-erbB-2/ear-1. *Cancer Res* **49**: 3104–3108

Tsuda H, Callen DF, Fukutomi T, Nakamura Y and Hirohashi S (1994) Allele loss on chromosome 16q24.2–qter occurs frequently in breast cancers irrespectively of differences in phenotype and extent of spread. *Cancer Res* **54**: 513–517

Wasmuth JJ, Park C and Ferrell RE (1989) Report of the committee on the genetic constitution of chromosome 5. *Cytogenet Cell Genet* **51**: 137–148

Winquist R, Hampton GM, Mannermaa A, Blanco G, Alavaikko H, Kiviniemi H, Taskinen PJ, Evans FA, Newsham I and Cavenee WK (1995) Loss of heterozygosity for chromosome 11 in primary human breast tumours is associated with poor survival after metastasis. *Cancer Res* **55**: 2660–2664

Wooster R, Bignell G, Lancaster J, Swift S, Seal S, Mangion J, Collins N, Gregory S, Gumbs C, Micklem G, Barfoot R, Hamoudi R, Patel S, Rice C, Biggs P, Hashim Y, Smith A, Connor F, Arason A, Gudmundsson J, Ficenec D, Kelsell D, Ford D, Tonin P and Stratton MR (1995) Identification of the breast cancer susceptibility gene BRCA2. *Nature* **378**: 789–792

© Cancer Research Campaign 1998